# Lidocaine promotes apoptosis in breast cancer cells by affecting VDAC1 expression

**DOI:** 10.1186/s12871-022-01818-y

**Published:** 2022-08-30

**Authors:** Dingde Long, Xingjun Fang, Peihua Yuan, Liqin Cheng, Hongtao Li, LiangChao Qu

**Affiliations:** 1 Department of Anesthesiology, Medical Center of Anesthesiology and Pain, Jiangxi Province, the First Affiliated Hospital of Nanchang University, No. 17, Yong Wai Zheng Road, Donghu district, 330000 Nanchang, P. R. China; 2 Department of Physiology and Biophysics, School of Medicine, Virginia Commonwealth University, Richmond, VA USA

**Keywords:** Lidocaine, Voltage-dependent anion channel 1, Invasive breast cancer, Apoptosis

## Abstract

**Objective:**

To investigate the effect of lidocaine on the expression of voltage-dependent anion channel 1 (VDAC1) in breast invasive carcinoma (BRCA) and its impact on the apoptosis of breast cancer cells.

**Methods:**

We collected clinical data from patients with invasive breast cancer from 2010 to 2020 in the First affiliated hospital of Nanchang University, evaluated the prognostic value of VDAC1 gene expression in breast cancer, and detected the expression of VDAC1 protein in breast cancer tissues and paracancerous tissues by immunohistochemical staining of paraffin sections. Also, we cultured breast cancer cells (MCF-7) to observe the effect of lidocaine on the apoptosis of MCF-7 cells.

**Results:**

Analysis of clinical data and gene expression data of BRCA patients showed VDAC1 was a differentially expressed gene in BRCA, VDAC1 may be of great significance for the diagnosis and prognosis of BRCA patients. Administration of lidocaine 3 mM significantly decreased VDAC1 expression, the expression of protein Bcl-2 was significantly decreased (*p* < 0.05), and the expression of p53 increased significantly (*p* < 0.05). Lidocaine inhibited the proliferation of MCF-7 breast cancer cells, increased the percentage of G2 / M phase cells and apoptosis.

**Conclusion:**

Lidocaine may inhibit the activity of breast cancer cells by inhibiting the expression of VDAC1, increasing the apoptosis in breast cancer cells.

**Supplementary Information:**

The online version contains supplementary material available at 10.1186/s12871-022-01818-y.

## Introduction

Breast cancer is one of the most common malignancies in women [1, 2]. According to the American Cancer Society report, breast cancer accounts for more than 29% of women's newly diagnosed cancers and has become the leading cause of cancer-related deaths in women aged 20 to 59 years [3]. Breast invasive carcinoma (BRCA) is a malignant tumor in which cancer cells have penetrated the basement membrane of the Breast catheter or lobular acini and invaded the stroma. Infiltrating ductal carcinoma is the most common type of BRCA found in more than 85% of BRCA and has a poor prognosis [4–6]. Retrospective studies showed that Anesthesia affects the survival of cancer patients [7], regional anesthesia may affect cancer recurrence or a reduced risk of cancer recurrence [8, 9]. One possible reason for improved cancer outcomes by regional anesthesia is that local anesthetic is associated with antitumor effects, including prevention of cancer cell proliferation, migration, or invasion [10, 11]. Perioperative intravenous lidocaine has been shown to reduce postoperative pain and opioid requirements [12, 13]. Lidocaine can also induce apoptosis and inhibit tumor growth in human breast tumor cells and others in vitro [14], but the specific mechanism is unclear.

Voltage-dependent anion channels (VDACs) are a kind of porous ion channel proteins located on the mitochondrial outer membrane of a cell [15]. VDAC allows the diffusion of small hydrophilic molecules that play an essential role in regulating mitochondria's transmembrane and energy metabolism by transporting ions and molecules (e. g., ATP, ADP, pyruvate, malate, and other metabolites) [16].In vivo and in vitro studies demonstrated the involvement of VDAC in tumor progression [17, 18]. There are three highly homologous isotypes of VDACs in mammals: VDAC1, VDAC2, and VDAC3, where VDAC1 ubiquitously expresses [19] in all tissue types. VDAC1 and the complex of glycolytic hexokinase regulate metabolites that provide a metabolic advantage to cancer cells through the outer membrane channels [20]. VDAC1 is regarded as a potential target for anti-cancer therapy [18, 20].

As a local anesthetic, the mechanism of action of lidocaine is by blocking voltage-gated sodium channels leading to reversible blockade of action potential propagation. Lidocaine effectively inhibits the invasiveness of cancer cells at concentrations used in surgical procedures, and it have been shown to trigger apoptosis in a variety of human cells [21]. However, the mechanisms underlying these effects are not yet fully understood. In this study we used clinical data of invasive breast cancer patients, high-throughput gene expression data, and in vitro experiments to investigate the effect of lidocaine on VDAC1 expression in BRCA and its mechanism.

## Material and methods

### Ethics

The research was approved by the Ethics Committee of our institution and was in agreement with the Declaration of Helsinki. Written informed consent was obtained for all patients.

### Clinical data collection

Clinical data related to invasive breast cancer patients from 2010 to 2020 were collected from the First affiliated hospital of Nanchang University, including age, sex, pathological N stage, T stage, M stage, and whether metastasis occurred, whether recurrence and postoperative survival follow-up status. High-throughput genomic analysis techniques collected high-throughput gene expression data from tissue samples of invasive breast cancer.

### Immunohistochemistry

Paraffin sections of BRCA tissue and normal breast tissue were baked in a 60℃ incubator for 120 min, followed by treatment in 0.3% H2O2 for 30 min for dewaxing and hydration, then incubated with VDAC1 (purchased from Cell Signaling Technology, USA) in PBS at 1:100 to 1:150 and overnight at 4℃. They were then treated with a 1:200 concentration of cast iron acylation secondary antibodies (Santa Cruz Biotechnology, Santa Cruz) for 30 min at 37℃, dehydrated and transparent, then blocked with neutral resin and covered with glass slides. Subsequently, they were observed by high magnification under a light microscope.

### Cell culture and viability assay

Breast cancer cells MCF-7 cells (ATCC, USA) were cultured in RPMI-1640 medium containing 1% penicillin and streptomycin and 10%FBS. Cells were incubated at 37 °C at 95% humidity and 5%CO2 environment. The RPMI-1640 medium was replaced every 2 days. The MTT method determined cell viability by setting MCF-7 cells in 96-well plates for 24 h and incubating them with lidocaine. Lidocaine was added at 0 mM, 1 mM, and 3 mM differentiate, and cell activity was measured after incubation. Cells of each group were grown for 24 h at 37℃. After incubation, MTT was added to each culture well at a final concentration of 2 mg/ml, and the cells were incubated at 37 C for 4 h. Then, 100uL of dimethyl sulfoxide was added and measured the absorbance of 570 nm.

### Immunofluorescence

MCF-7 cells were plated in 24-well plates, fixed with 0.1% Triton at 4℃ for 30 min, washed in PBS, treated with 5% bovine serum albumin for 60 min at room temperature, and then stained with a primary antibody to VDAC1 at 4℃ overnight. Secondary antibody FITC (goat anti-mouse, AbCAM) was diluted to 1:200 in PBS and incubated at 4 C for 60 min, and Coverslips were then loaded onto slides with 1% 4 ′ 6-diamidine-2 ′ -phenylindole dihydrochloride (DAPI) for 10 min. Cell images were visualized using a scanning confocal fluorescence microscope (Olympus Co, Japan).

### Western blot

The MCF-7 cells were incubated for 24 h. It was divided into three groups: the control group (culture medium), lidocaine 1 mM, and lidocaine 3 mM group. Supernatants were collected from lysed cells in lysis buffer. Protein samples were separated by SDS-PAGE and then electrotransferred to a polyvinylidene difluoride (cellulose nitrate) membrane. Membranes were then incubated with 5% skim milk for 2 h and then with primary antibody (anti-Bcl-2, anti-p53) overnight at 4 °C. Horseradish peroxidase-conjugated secondary antibody was used. Bands were detected by HRP chemiluminescence. The Gel image analysis system caught protein blotted band grayscale. The ratio of Bcl-2, p53, and β-actin was determined and calculated.

### Flow cytometry for cell cycle analysis

The MCF-7 cells were synchronized by serum starvation for 24 h prior to treatment with lidocaine. Cells treated with lidocaine were digested with trypsin. 70% alcohol was used to fix the cells on ice for at least 2 h, followed by centrifugation at 1500 RPM (revolutions per minute) for 10 min. The cell precipitate was resuspended with 500 µl guava cell cycle reagent. After incubation for 30 min at 37 °C in the dark, cell cycle distribution was analyzed by BD FACSMelody™ flow cytometry.

### Flow cytometry for apoptosis analysis

The lidocaine-treated MCF-7 cells were digested with trypsin, centrifuged at 300 g for 5 min, the supernatants were discarded, the cells were collected, washed again with PBS, and then gently resuspended and counted. 5 × 10^5^ cells were collected and centrifuged at 300 g for 5 min, and the supernatants were discarded. The cells were resuspended with PBS again. After centrifugation, the supernatants were discarded. The cells were resuspended with 100 ul diluted 1 × Annexin V Binding Buffer. 2.5 ul Annexin V-FITC and 2.5 ul propidium iodide (PI) staining were then added. The samples were incubated for 15 min in the dark at room temperature. 400ul diluted 1 × Annexin V Binding Buffer were then added to mix the samples, apoptosis was analyzed by BD FACSMelody™ flow cytometry.

### Statistical analysis

The results are reported as the mean ± SD. Student’s t-test, one-way analysis of variance (ANOVA), and two-way ANOVA followed by Bonferroni’s multiple comparisons test were used for the statistical analyses and were performed with Prism 7 (GraphPad Software). The survival analysis and univariate analysis were performd with SPSS software, version 19.0(SPSS, INC, USA). *P* < 0.05 were considered statistically significant.

## Results

### Clinical significance of VDAC1 gene expression in patients with invasive breast cancer

The correlation between VDAC1 expression and clinicopathological characteristics in BRCA patients(Table 1), All cases were divided into high expression group and low expression group according to the median VDAC1 gene expression. There was no significant differences in VDAC1 expression in terms of age, gender, pathological N stage, and chemotherapy was received in BRCA patients (*p* > 0.05), and significant differences in pathological stage, T stage, M stage and whether metastasis occurred in BRCA patients (*p* < 0.05). Univariate regression analysis showed that (Table 2) Survival in BRCA patients was independent of gender and race (*p* > 0.05) and significantly correlated with age, pathological stage, receipt of chemotherapy, metastasis, and VDAC1 expression (*p* < 0.05).

**Table 1:** The correlation between VDAC1 expression and clinicopathological characteristics in BRCA patients

Characteristics	BRCA cohort	VDAC1 expression		*P*
	(*n* = 527)	Low (*n* = 263)	High (*n* = 264)	
Age (median)	59 (48–67)	57 (47–65)	60 (50–68)	0.053
Gender				
Male	6 (1.1)	2	4	0.414
Female	521 (98.9)	261	260	
Pathologic stage				
i	94 (17.8)	62	32	0.001
ii	296 (56.2)	144	152	
iii	111 (21.1)	50	61	
iv	26 (4.9)	7	19	
Pathology T stage				
T1	134 (25.4)	81	53	0.027
T2	313 (59.4)	148	165	
T3	60 (11.4)	27	33	
T4	20 (3.8)	7	13	
Pathology N stage				
N0	266 (50.5)	143	123	0.198
N1	171 (32.4)	83	88	
N2	61 (11.6)	26	35	
N3	29 (5.5)	11	18	
Pathology M stage				
M0	512 (97.2)	260	252	0.019
M1	15 (2.8)	3	12	
Radiation therapy				
No	262 (49.7)	121	141	0.089
Yes	265 (50.3)	142	123	
Metastasis				
No	212 (40.2)	119	93	0.019
Yes	315 (59.8)	144	171

**Table 2:** Univariate regression analysis of BRCA patient survival

Characteristics	Hazard Ratio	95% confidence interval	*P*
Age	1.024	1.007–1.042	0.006
Gender	1.048	0.137–1. 502	0.456
Pathologic_stage	1.933	1.509–2.475	< 0.001
Pathology_T_stage	1.496	1.136–1.971	0.004
Pathology_N_stage	1.496	1.182–1.894	0.001
Pathology_M_stage	1.097	1.053–1.186	< 0.001
Radiation_therapy	0.329	0.208–0.522	< 0.001
Metastasis	2.415	1.481–3.938	< 0.001
VDAC1	1.235	1.007–1.539	0.041

### The expression of VDAC1 in BRCA

**Fig. 1:**
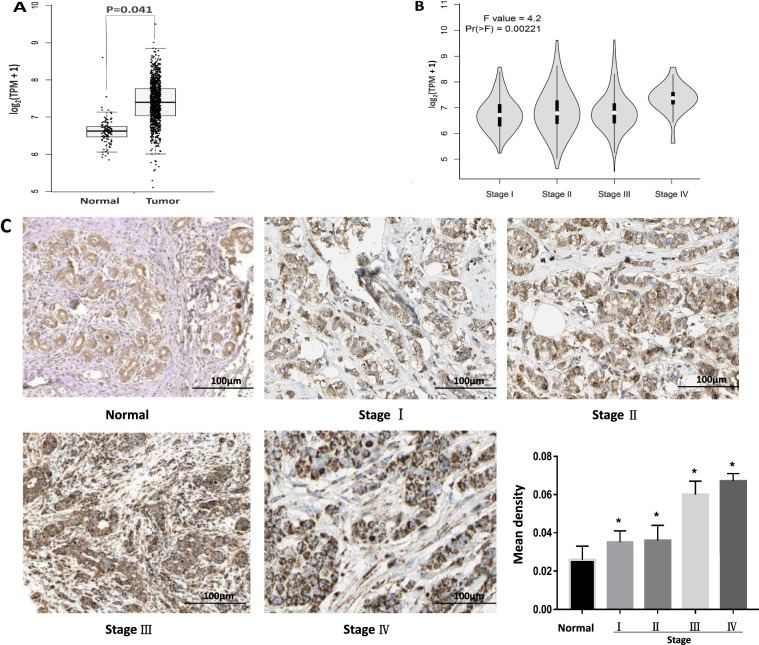
Expression of VDAC1 in BRCA. **A** Expression of the VDAC1 gene in normal breast tissues and BRCA; **B** Expression of the VDAC1 gene in different pathological stages of BRCA; **C** Expression of VDAC1 protein in BRCA tissues at different stages of tumor growth and adjacent cancerous tissues. Compared with the Normal group, * *P* < 0.05

VDAC1 gene expression was significantly higher in BRCA than in normal breast tissue which from the same patients. (Fig. 1A, *p* = 0.0041). Next, VDAC1 expression in the BRCA tumor stages was further analyzed. The results showed that the expression level of VDAC1 varied significantly in the different stages of BRCA tumors (Fig. 1B, *p* = 0.022). and the results of post hoc tests by Bonferroni’s multiple comparisons test showed that the expression level of VDAC1 varied significantly only in stage I and stage IV (*p* = 0.046). The expression of VDAC1 protein In breast cancer tissues and adjacent tissues was performed by immunohistochemical staining of paraffin sections. The results showed that VDAC1 protein expression was significantly higher in breast cancer tissues than normal (Fig. 1C *p* < 0.003).

### Association between VDAC1 gene expression and prognostic survival in patients with invasive breast cancer

**Fig. 2:**
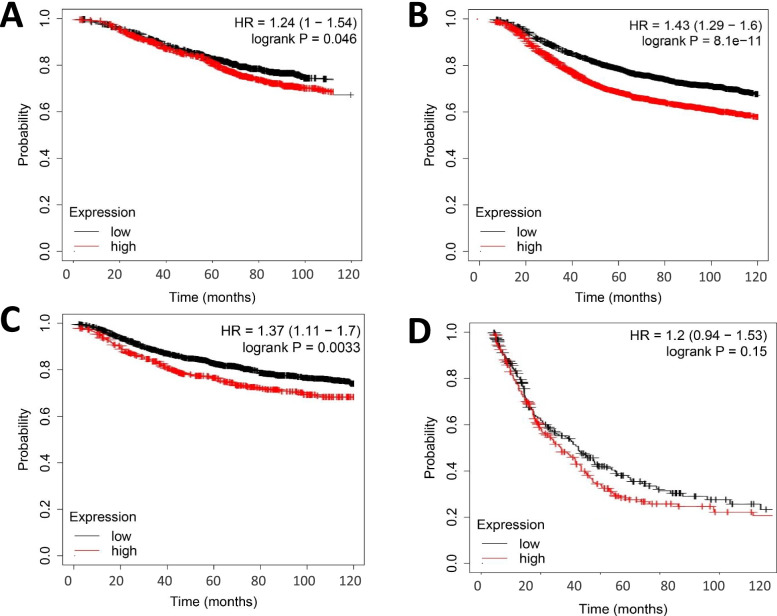
Relationship between VDAC1 gene expression and the prognostic survival of BRCA patients. **A** Association between gene levels of VDAC1 and overall survival of BRCA. **B** Association between gene levels of VDAC1 and recurrence-free survival of BRCA. **C** Association between gene levels of VDAC1 and survival without distant metastasis in BRCA. **D** Association between gene levels of VDAC1 and post-progression survival of BRCA

To determine the prognostic value of gene expression of VDAC1 in patients with BRCA, we further analyzed the survival status of both high and low expression. The results showed that the gene level of VDAC1 was significantly correlated with overall survival (OS) (Fig. 2A, *p* < 0.05), recurrence-free survival (RFS) (Fig. 2B, *p* < 0.05), distant metastasis-free survival (DMFS) (Fig. 2C, *p* < 0.05). However, the gene expression level of VDAC1 was not correlated with post-progression survival (PPS)(Fig. 2D, *p* > 0.05). Combined with the expression of different VDAC1 genes in tumors and normal tissues, these results suggest that the gene expression levels of VDAC1 may be necessary for the survival of BRCA patients.

### Liddocaine promotes apoptosis of MCF-7 cells by inhibiting VDAC1 expression

**Fig. 3:**
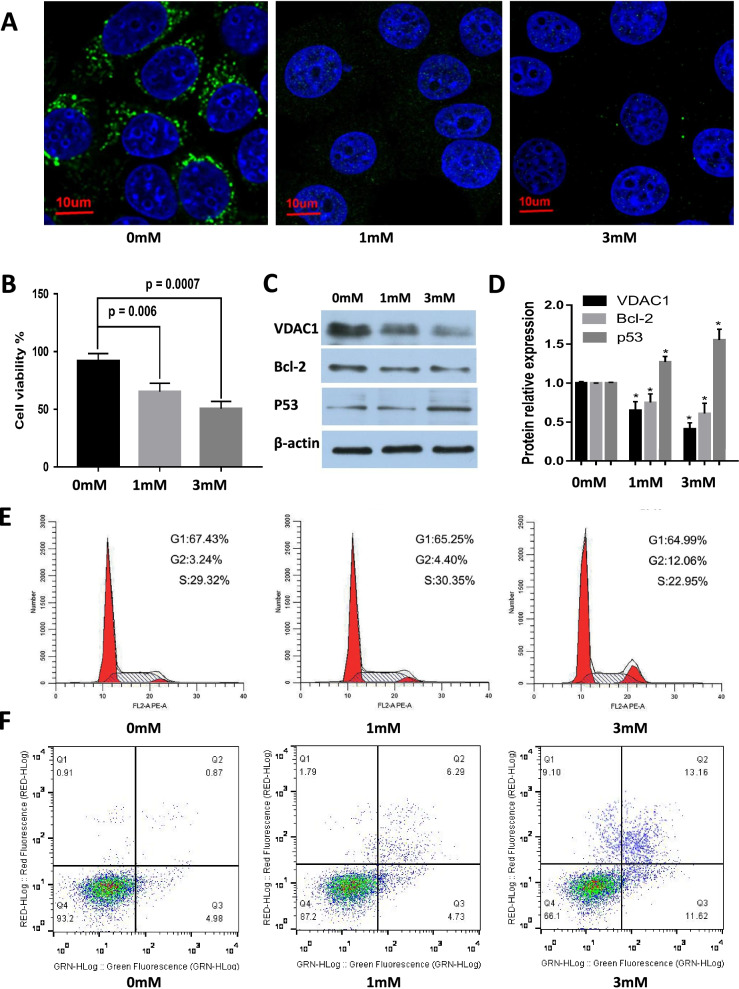
Lidocaine promotes apoptosis of MCF-7 cells by inhibiting VDAC1 expression. **A** VDAC1 expression in different concentrations of MCF-7 cells was detected by immunofluorescence; **B** Effect of different concentrations of lidocaine on the activity of MCF-7 cells; **C** and **D** Western Blot measured the expression of VDAC1 and the expression of the pro-apoptotic protein Bcl-2 and the tumor suppressor gene p53 in different concentrations of lidocaine MCF-7 cells; **E** The cell cycle distribution of MCF-7 cells treated with various concentrations (0,1,3 mM) of lidocaine; **F** Lidocaine induced apoptosis in MCF-7 cells measured by flow cytometry. Compared with the 0 mM lidocaine group, * *P* < 0.05

The VDAC1 expression of MCF-7 cells was detected by immunofluorescence (green fluorescence), and the expression of VDAC1 was significantly reduced in MCF-7 cells cultured at 1 mM and 3 mM lidocaine after 24 h at 37℃ (Fig. 3A); The MTT assay shows that 1 mM (0.6537 ± 0.0727, *P* = 0.006, *n* = 3) and 3 mM (0.5055 ± 0.0638, *P* = 0.0007, *n* = 3) lidocaine can significantly inhibit MCF-7 cell activity compared with the control group 0 mM lidocaine (Fig. 3B), Compared with control groups, lidocaine was able to significantly inhibit VDAC1 expression in MCF-7 cells, with lidocaine at 1 mM (0.65 ± 0.11, *P* = 0.004, *n* = 3) and 3 mM (0.41 ± 0.08, *P* = 0.0002, *n* = 3). Meanwhile, the apoptotic Bcl-2 protein was inhibited upon treated of lidocaine, with 1 mM (0.75 ± 0.11, *P* = 0.04, *n* = 3) and 3 mM (0.61 ± 0.13, *P* = 0.006, *n* = 3), but the expression of the tumor suppressor gene p53 increased with the increasing concentration of lidocaine, with 1 mM (1.27 ± 0.07, *P* = 0.02, *n* = 3) and 3 mM (1.55 ± 0.14, *P* = 0.007, *n* = 3) (Fig. 3C and D). Flow cytometry was used to measure the effects of lidocaine at various doses of 0, 1, and 3 mM on the cell cycle distribution of MCF-7, the proportion of MCF-7 cells in the G2/M phase was 12.06% in 3 mM group, respectively, which was signifi cantly higher than that of the control group (3.24%). The effect of lidocaine on apoptosis of MCF-7 cells also measured by flow cytometry. As shown in Fig. 3F, lidocaine treatment significantly induced apoptosis of MCF-7 cells.

## Discussion

Lidocaine has analgesic, antihyperalgesic, and anti-inflammatory effects [12]. Studies have shown that intravenous lidocaine as a component of multimodal analgesia can reduce opioid demand and improve pain relief [13]. In a small clinical trial in patients with breast cancer surgery, lidocaine infusion did not significantly affect postoperative pain [22]. Although the antitumor mechanisms of lidocaine are unknown, the use of lidocaine during surgery may be beneficial in limiting surgery-induced cancer cells escaping from dormancy or cell shedding [7]. In vitro experiments in this study showed that 1 mM lidocaine significantly inhibited breast cancer cell activity in vitro, decreased Bcl-2 protein expression, and promoted the expression of tumor suppressor gene p53. Flow cytometry showed that lidocaine inhibited cell cycle in G2/M phase and promoted cell apoptosis. But the mechanism by which lidocaine suppresses the activity of breast cancer cells is poorly understood currently.

Cancer cells are characterized by a higher proliferative state than normal cells, with changing cell metabolism, and mitochondria (the site of ATP generation) are closely related to cell metabolism [23]. Studies have confirmed that mitochondria's bioenergy, biosynthesis, and signal transduction are involved in tumor occurrence and development [24]. Metabolites and ions are exchanged through the outer mitochondrial membrane between the cytoplasm and the mitochondria. The mitochondrial porin VDAC controls the process. VDAC1 is one of the most studied members of the VDAC protein family and is located on the outer mitochondrial membrane [25]. VDAC1 acts as a gatekeeper to mitochondria and controls the exchange of metabolites between the cytoplasm and the mitochondria, fatty acid ions, calcium ions, reactive oxygen species, and cholesterol [26]. Furthermore, VDAC1 interacts with many cytoplasm proteins, endoplasmic reticulum, and mitochondria to regulate metabolic processes, cell proliferation, and apoptosis [27]. However, the specific biological functions and regulatory mechanisms of VDAC1 in BRCA are still unclear.

With the extensive investigation of genomic changes in cancer cells, identifying subgroups of patients with different prognoses and different treatment responses offers the possibility of finding new drug targets [28–30]. To explore the clinical significance of VDAC1 in BRCA, the research group finally extracted and integrated the data of 527 clinical samples through clinical date and High-throughput data on gene expression of BRCA patients. The results showed that the level of VDAC1 expression significant differences in pathological stage, T stage, M stage, and whether metastasis occurred in BRCA patients, but not in age, sex, pathological N stage, or receiving chemotherapy. The results of univariate regression analysis showed that survival in BRCA was not related to gender and race. It was significantly correlated with age, pathological stage, chemotherapy, metastasis, and VDAC1 expression level. On the other hand, VDAC1, an essential protein of the mitochondrial outer membrane, may influence tumors' occurrence and development [24]. VDAC1 may be a potential target for drug therapy in BRCA.

In this study, we first explored the effect of VDAC1 on BRCA and its relationship with the Clinicopathological features of BRCA patients. The results showed that the expression of the VDAC1 gene in BRCA was significantly higher than in normal breast tissue, and the VDAC1 gene expression level also varied substantially in different pathological stages of BRCA tumors. Moreover, the expression level of the VDAC1 gene also varied considerably in different pathological stages of BRCA tumors. VDAC1 protein expression was substantially higher in BRCA tissues than in normal breast tissues by immunohistochemical staining of paraffin sections of clinical specimens. Predictive survival analysis showed that the gene levels of VDAC1 were significantly associated with overall survival, recurrence-free survival, and distant metastasis-free survival. Therefore, VDAC1, as a differentially expressed gene in BRCA tissues, may be essential for the diagnosis and prognostic survival of BRCA patients.

The aggressiveness of cancer cells is effectively inhibited at the concentration of lidocaine used in surgical procedures [31, 32]. This invasive effect is not related to its anesthetic activity (sodium channel blocking) [33]. However, the impact of lidocaine on anion channels is not fully understood. To explore the effect of lidocaine on VDAC1 expression in breast cancer cells, we analyzed VDAC1 expression after different concentrations of lidocaine cultures by immunofluorescence labeling staining. The results showed that 1 mM lidocaine was able to inhibit VDAC1 expression significantly. Meanwhile, as shown by the protein expression assay, the expression of VDAC1, the expression of apoptosis inhibitory protein Bcl-2 was reduced considerably, and the expression of tumor suppressor gene p53 was increased dramatically in MCF-7 breast cancer cells after 1 mM lidocaine culture. Flow cytometry showed that lidocaine promoted cell apoptosis in a concentration dependent manner and inhibited cell cycle in G2/M phase. Therefore, lidocaine may promote apoptosis in breast cancer cells by inhibiting VDAC1 expression from inhibiting breast cancer cell activity.

It was reported that, Lidocaine induces cell apoptosis via regulating MEK/ERK [34]and PI3K/AKT [35] signaling pathways. The VDAC1 protein, as a key regulator within the mitochondrial permeability transition pore complex, which is closely associated with Bcl-2 family proteins, and plays a role in mitochondria-mediated apoptosis. It was also reported that [36], VDAC1 negatively regulates the PI3K/Akt pathway through GSK3β.The mechanism of action of lidocaine as a local anesthetic is through a blockade of voltage-gated sodium channels leading to a reversible block of action potential propagation. Therefore, we believe that, firstly, lidocaine affects the alteration of ion channels and then the alteration of intracellular signaling pathways.

## Conclusion

In conclusion, VDAC1, as a differentially expressed gene in BRCA, may have important implications for the diagnosis and prognostic survival of BRCA patients. Meanwhile, VDAC1 may be a potential target for BRCA drug therapy. Lidocaine may promote apoptosis in breast cancer cells by inhibiting VDAC1 expression, thereby inhibiting breast cancer cell activity.

## Supplementary Information

**Additional file 1: Supplementary Figure 1. **The expression of VDAC1 in different concentrations of lidocaine MCF-7cells. **Supplementary Figure 2.** The expression of Bcl-2 in different concentrations of lidocaine MCF-7cells. **Supplementary Figure 3.** The expression of p53 in different concentrations of lidocaine MCF-7cells. **Supplementary Figure 4.** β-actin.

## Data Availability

The datasets generated and/or analysed during the current study are available in this paper.
