# Supplementary material for: Lidocaine promotes apoptosis in breast cancer cells by affecting VDAC1 expression
Source: BMC Anesthesiol. 2022 Aug 30;22:273. doi: 10.1186/s12871-022-01818-y (PMC9426218; doi:10.1186/s12871-022-01818-y)
Supplement: Supplementary file 1 — Additional file 1: Supplementary Figure 1. The expression of VDAC1 in different concentrations of lidocaine MCF-7cells. Supplementary Figure 2. The expression of Bcl-2 in different concentrations of lidocaine MCF-7cells. Supplementary Figure 3. The expression of p53 in different concentrations of lidocaine MCF-7cells. Supplementary Figure 4. β-actin. [file 12871_2022_1818_MOESM1_ESM.pdf]

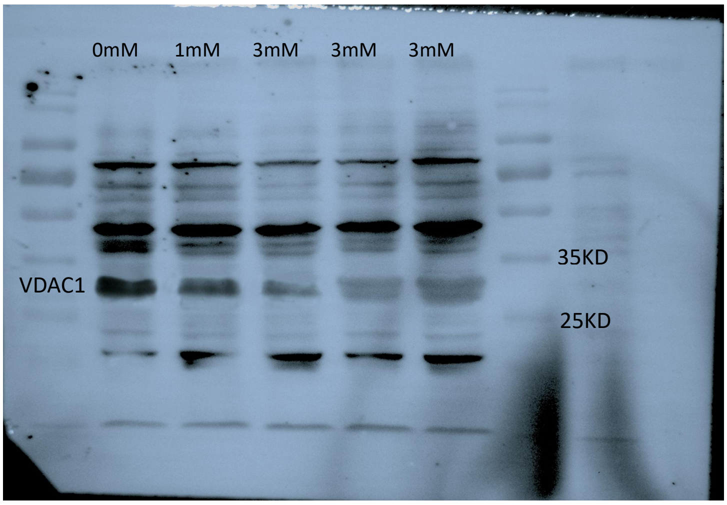

Supplementary Figure1, the expression of VDAC1 in different concentrations of lidocaine MCF-7 cells.

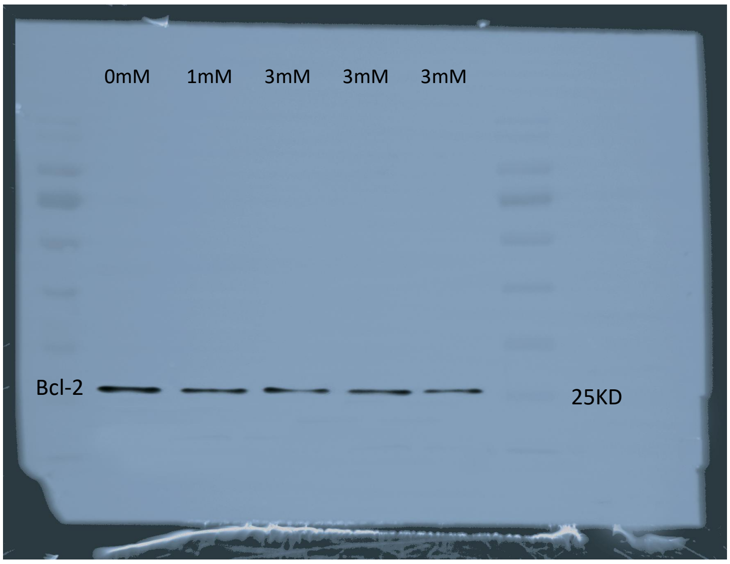

Supplementary Figure2, the expression of Bcl-2 in different concentrations of lidocaine MCF-7 cells.

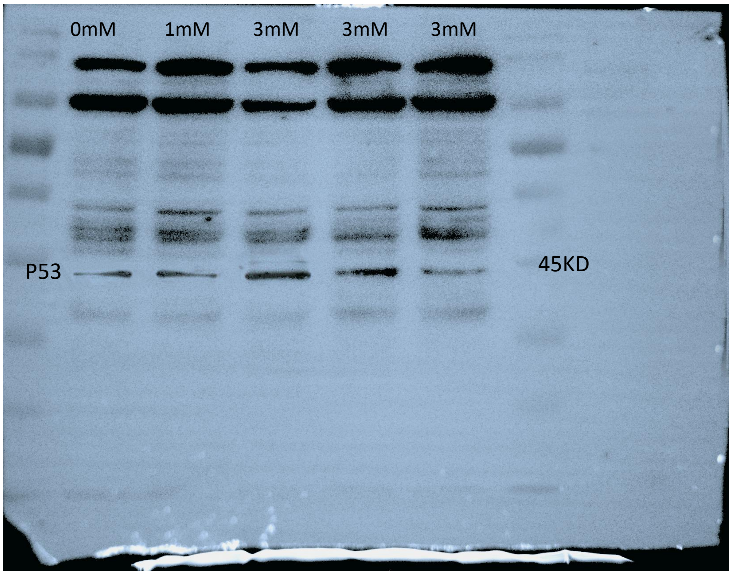

Supplementary Figure3, the expression of p53 in different concentrations of lidocaine MCF-7 cells.

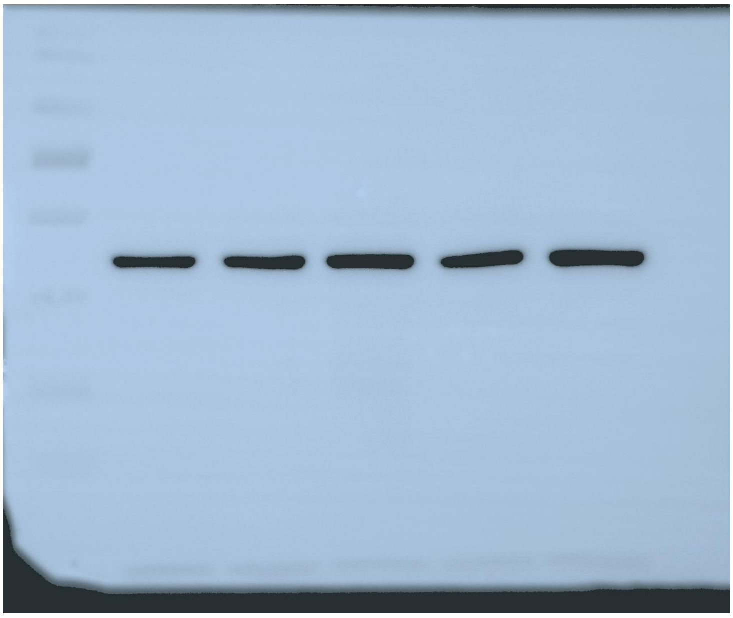

Supplementary Figure4, β-actin
